# Supplementary material for: The Systems Biology Research Tool: evolvable open-source software
Source: BMC Syst Biol. 2008 Jun 29;2:55. doi: 10.1186/1752-0509-2-55 (PMC2446383; doi:10.1186/1752-0509-2-55)
Supplement: Additional file 1 — SBRT Archive. An archive of the current version of the Systems Biology Research Tool. [file 1752-0509-2-55-S1.zip › sbrt-1.4.0/doc/developers_guide/api/sbrt/shell/io/class-use/MapTextOutputFile.html]

Uses of Interface sbrt.shell.io.MapTextOutputFile


|  |  |  |
| --- | --- | --- |
| **Overview** \| **Package** \| **Class** \| **Use** \| **Tree** \| **Deprecated** \| **Index** \| **Help** | |  |
| PREV   NEXT | **FRAMES**    **NO FRAMES**     **All Classes** |  |


---


## **Uses of Interface sbrt.shell.io.MapTextOutputFile**

| Packages that use MapTextOutputFile | |
| --- | --- |
| **sbrt.shell.io** | Provides classes and interfaces for reading and writing files. |
| **sbrt.shell.mng.fba** | Provides classes and interfaces for creating Flux Balance Analysis process managers. |

Uses of MapTextOutputFile in sbrt.shell.io

| Classes in sbrt.shell.io that implement MapTextOutputFile | |
| --- | --- |
| `class` | `AbstractMapOutputFile<K,V>`             This abstract class is a skeleton implementation of `MapTextOutputFile`. |
| `class` | `ComoFile<K,V>`             This class is used to write maps to text files in a column-oriented way. |
| `class` | `RomoFile<K,V>`             This class is used to write maps to text files in a row-oriented way. |

Uses of MapTextOutputFile in sbrt.shell.mng.fba

| Methods in sbrt.shell.mng.fba that return MapTextOutputFile | |
| --- | --- |
| `static MapTextOutputFile<java.lang.String,Interval>` | `RandomConstraintsManager.getOutputFile(ManagerInput<java.lang.String,java.lang.String> input, java.io.PrintWriter out, IrrevRxnFluxome fluxome)`             Returns an empty output file that is suitable for storing multiple sets of flux constraints. |
| `static MapTextOutputFile<java.lang.String,Interval>` | `RandomEnvManager.getOutputFile(ManagerInput<java.lang.String,java.lang.String> input, java.io.PrintWriter out, IrrevRxnFluxome fluxome)`             Returns an empty output file that is suitable for storing multiple environments. |
| `static MapTextOutputFile<java.lang.String,Interval>` | `RandomConstraintsManager.getOutputFile(java.lang.String fileName, FileFormat format, java.util.Set<java.lang.String> headers, IrrevRxnFluxome fluxome)`             Returns an empty output file that is suitable for storing multiple sets of flux constraints. |
| `static MapTextOutputFile<java.lang.String,Interval>` | `RandomConstraintsManager.getOutputFile(java.lang.String fileName, java.util.Set<java.lang.String> headers, IrrevRxnFluxome fluxome)`             Returns an empty output file that is suitable for storing multiple sets of flux constraints. |

---


|  |  |  |
| --- | --- | --- |
| **Overview** \| **Package** \| **Class** \| **Use** \| **Tree** \| **Deprecated** \| **Index** \| **Help** | |  |
| PREV   NEXT | **FRAMES**    **NO FRAMES**     **All Classes** |  |


---
